# Transactions of the Thirty-Sixth Annual Meeting of the Medical Society of Virginia

**Published:** 1860-06

**Authors:** 


TRANSACTIONS OF THE THIRTY-SIXTH ANNUAL MEETING OF THE MEDICAL SOCIETY OF VIRGINIA: Held in April, 1859.

This is a pamphlet of 22 pages, containing the simple record of proceedings of the Annual Meeting, and the Address of the President of the Society, Prof. Levin S. Joynes, of Richmond, Va. The address is well written and may be read with interest and profit by all. The design of its author was, doubtless, to awaken more interest in the State Society, on the part of the physicians of that State.

In the record of proceedings is a copy of the bill for establishing a *State Record of Medical Examiners*, which was adopted by the Society and recommended to the State Legislature.

It is well known, that we have been for many years in favor of the establishment of similar Boards in every State in the Union, by which all candidates for admission into the profession should be examined whether they possessed a diploma from a Medical College or not. Such a measure, properly executed, would speedily place every Medical College where it should be, strictly on its *merits* as an institution for imparting sound medical instruction.

